# Metastatic melanoma: prognostic factors and survival in patients with brain metastases

**DOI:** 10.1007/s11060-017-2591-9

**Published:** 2017-08-17

**Authors:** E. Frinton, D. Tong, J. Tan, G. Read, V. Kumar, S. Kennedy, C. Lim, R. E. Board

**Affiliations:** 1 University of Manchester, Manchester, UK; 2 Lancashire Teaching Hospitals NHS Trust, Royal Preston Hospital, Preston, PR2 9HT UK

**Keywords:** Metastatic melanoma, Brain metastases, Survival, Radiotherapy

## Abstract

Brain metastases from malignant melanoma carry a poor prognosis. Novel systemic agents have improved overall survival (OS), but the value of whole-brain radiotherapy (WBRT) and stereotactic radiosurgery (SRS) remains uncertain. The melanoma-specific graded prognostic assessment (msGPA) provides useful prognostic information, but the relevance to the modern-day population has not been validated. Since 2011, 53 patients received treatment for brain metastases from malignant melanoma at the Rosemere Cancer Centre medical oncology clinic. Data were collated on demographic factors and survival. Survival analyses were performed using Kaplan–Meier methods. Cox regression was used to identify prognostic factors on univariate and multivariate analysis. OS from the date of diagnosis of brain metastases was 4.83 months (range 0.27–30.4 months). On univariate analysis, BRAF, performance status and msGPA were significant prognostic indicators for OS (p = 0.0056, p = 0.0039 and p = 0.0001 respectively). msGPA remained significant on multivariate analysis (p = 0.0006). OS for BRAF-positive patients receiving targeted treatment (n = 22) was significantly better than for BRAF-negative patients (n = 26), with median survival times of 8.2 and 3.7 months respectively (p = 0.0039, HR 2.36). SRS combined with systemic agents (n = 16) produced an OS of 13.5 months. Patients receiving WBRT alone (n = 21) had a poor prognosis (2.2 months). The msGPA remains a valid prognostic indicator in the era of novel systemic treatments for melanoma. BRAF-positive patients receiving targeted agents during their treatment had favorable survival outcomes. WBRT alone should be use with caution in the active management of melanoma brain metastases.

## Background

Malignant melanoma is the fifth most common cancer in the UK. Although the majority of patients present with early stage, operable disease, up to 20% have metastases at presentation [1]. Brain metastases occur in approximately 44% patients with metastatic melanoma, with a median overall survival (OS) of just 4 months [2]. Traditionally, brain metastases from melanoma have been deemed incurable by systemic therapy. Poor response rates to chemotherapy are most likely due to low drug concentrations accessing malignant cells owing to the protective nature of the blood–brain barrier [1]. There is evidence for the efficacy of novel systemic agents, both targeted BRAF inhibition and immunotherapy, in brain metastases from melanoma [1, 3–5]. Local treatment options include neurosurgery and radiotherapy. The latter is available for administration in two different forms; stereotactic radiosurgery (SRS) for patients with low-volume, low count brain metastases [6], or whole brain radiotherapy (WBRT) for patients with more widely disseminated intracranial disease. The evidence base for the efficacy of WBRT in the treatment of melanoma brain metastases is inconclusive. Many studies have shown no improvement in OS [7–9]. It has been hypothesised that this may be due to the resistant nature of melanoma cells to non-SRS radiation, having some ability to repair themselves following radiation insult [9, 10]. Other analyses suggest that WBRT may provide some benefit to intracranial control, particularly when used in combination with SRS and when there is stable extracranial disease [11]. In the absence of definitive guidance, clinicians can utilise a variety of prognostic tools to inform treatment decisions. The melanoma-specific Graded Prognostic Assessment (msGPA) [12] is one such tool that allocates patients into four categories according to number of brain metastases and their Karnofsky performance status (KPS). Patients with a higher msGPA score have a better OS [12]. The relevance of this tool to the modern day patient population, with its access to novel systemic therapies, has not been validated. One criticism of the msGPA is that it does not take into account patient age, presence of extracranial disease, leptomeningeal disease, aggregate brain tumour volume or BRAF status, all of which may be potential important prognostic indicators [2, 4, 13–15].

We aim to assess the validity of the msGPA in the modern day patient population. Important prognostic factors that influence the survival of patients with brain metastases from melanoma will be identified. The efficacy of SRS combined with other systemic therapies versus WBRT will be assessed.

## Methods and materials

Patients undergoing treatment for brain metastases in metastatic melanoma at the Rosemere Cancer Centre, within the Lancashire and South Cumbria Cancer Network (LSCCN), seen in the medical oncology clinic between 2011 and 2016 were identified (n = 57). The hospital database system was used to obtain clinic letters, scan results, BRAF testing outcomes and treatment regimes. The information was collated using Microsoft Excel (2010) software. When exact dates were unavailable, the 15th of the month was used. BRAF-mutant positive patients (n = 4) who presented prior to the availability of BRAF inhibitors were excluded to enable the efficacy of novel targeted therapies to be assessed.

Treatment of intracranial and extracranial disease was determined by BRAF status, symptoms including performance status, previous treatment, number of brain metastases, patients’ preferences and availability and funding of drugs as determined in the UK by the National Institute for Health and Care Excellence and the UK National Cancer Drugs Fund. In 2011 single agent BRAF inhibition was available in the UK as first line treatment, and subsequently in combination with MEK inhibition in July 2016. Second line ipilimumab was available in 2011 and first line in 2014. PD1 antibodies were available from the end of 2015 in both untreated and previously treated patients. Combination immunotherapy was not available during the time period of this audit nor were any clinical trials available for patients with brain metastases. Patients’ history and radiology were discussed at both a specialist skin and brain multidisciplinary team meeting to determine suitability for neurosurgery and radiotherapy including stereotactic treatment. Generally, patients with multiple brain metastases from BRAF positive melanoma would be offered BRAF inhibition (±MEK inhibition) as first line, followed by either systemic immunotherapy or whole brain radiotherapy second line. Patients with BRAF negative melanoma would be assessed for surgery and/or stereotactic radiotherapy as first line treatment and then offered systemic immunotherapy if available and not received previously. Patients ineligible for surgery or stereotactic radiotherapy and those patients who had exhausted previous systemic treatment options were assessed for whole brain radiotherapy.

StatsDirect [16] software was used to perform survival analysis according to BRAF status, msGPA score and radiotherapy modality. Kaplan-Meier (KM) curves [17] were generated and tested for statistical significance using peto’s log rank [18] technique. Where confidence intervals (CI) are quoted, Andersens 95% CI are used. Survival is quoted in months, standardised to a 30 day period to allow comparison.

Univariate and multivariate analysis were performed using Cox regression [19]. Prognostic factors tested included age, sex, BRAF status, KPS, msGPA, presence of extra cranial disease and presence of neurological symptoms. Factors that showed statistical significance (p < 0.05) on univariate testing were run in multivariate analysis in various combinations to determine if these associations maintained statistical significance when other factors were taken into account.

## Results

In total, N = 53 patients were eligible for analysis following exclusion of BRAF-mutant positive patients who did not receive targeted inhibition. For survival analysis according to BRAF status, a further five patients were excluded due to unknown BRAF status. Basic demographic and clinical covariate data is shown in Table 1.

**Table 1:** Patient demographics

Total number of patients	53
Age, median (range)	61 (20–85)
Sex, n (%)	
Male	31 (58)
Female	22 (42)
BRAF status, n (%)	
Positive	25 (47)
Negative	22 (42)
Unknown	5 (9)
Brain metastases present at initial diagnosis of metastatic melanoma, n (%)	22 (42)
Brain metastases symptomatic, n (%)	41 (77)
Number of brain metastases, n (%)	
1	15 (28)
2–3	10 (19)
>3	28 (53)
KPS, n (%)	
90–100	24 (45)
70–80	21 (40)
<70	8 (15)
msGPA score, n (%)	
0–1	21 (40)
2	12 (23)
3	6 (11)
4	14 (26)
Extra cranial disease presence, n (%)	42 (79)
Treatment for brain metastases, n (%)	
Total Neurosurgery	9 (17)
Neurosurgery alone	3 (6)
Total SRS	8 (15)
SRS alone	3 (6)
Total WBRT	23 (43)
WBRT alone	17 (32)
Total Immunotherapy	9 (17)
Immunotherapy alone	2 (4)
Total Targeted BRAF inhibitor	14 (26)
Targeted BRAF inhibitor alone	8 (15)
Combination therapy	15 (28)
Neurosurgery + WBRT	2 (4)
Neurosurgery + targeted BRAF inhibitor	4 (8)
Neurosurgery + SRS + immunotherapy	2 (4)
SRS + immunotherapy	3 (6)
WBRT + targeted BRAF inhibitor	2 (4)
WBRT + immunotherapy	2 (4)
Best supportive care	5 (9)

**Fig. 1:**
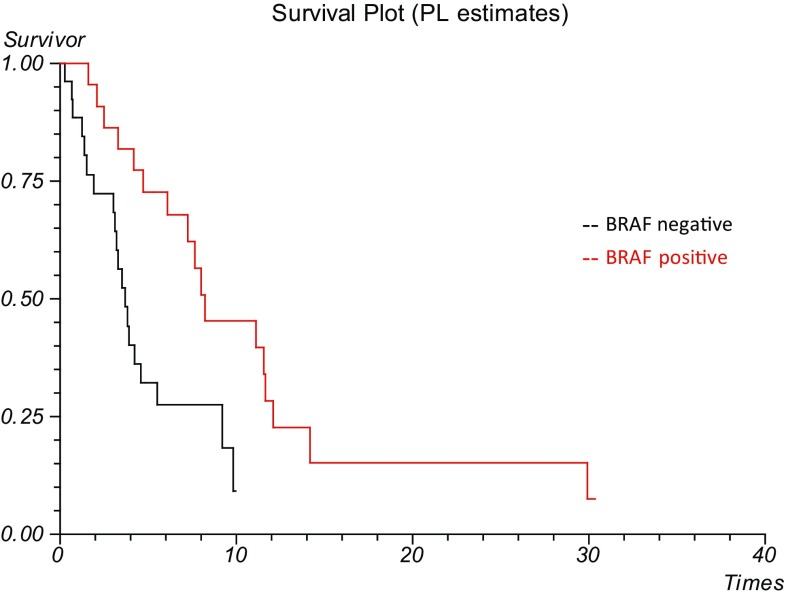
Overall survival from date of brain metastases diagnosis according to BRAF status. Better survival (p = 0.0039) in the BRAF-positive treated subgroup (n = 25), with a median OS of 8.23 months (95% CI 3.62–12.84) in comparison to 3.7 months (95% CI 2.78–4.62) for the BRAF-negative patients (n = 22)

Median OS from diagnosis of brain metastases to death was 4.83 months (range 0.27–30.4 months). BRAF-mutant positive patients had significantly better survival times than the BRAF-mutant negative group, at 8.23 month median OS (95% CI 3.62–12.84) and 3.7 months (95% CI 2.78–4.62) respectively (p = 0.0039) from the time of diagnosis of brain metastases (Fig. 1). N = 17 (32%) patients were receiving either immunotherapy or BRAF inhibition at the time of their brain metastases diagnosis. The median number of cycles of systemic treatment until development of brain metastases was 5 (range = 1–21). N = 6 patients (35%) continued on the same systemic treatment after their brain metastases were diagnosed. The remainder were switched to an alternative.

SRS was often given in combination with other treatments such as surgery and immunotherapy. N = 14 (26%) patients were treated with SRS for their brain metastases, either initially (n = 9) or on progression (n = 5). N = 10 (71%) patients demonstrated response to this treatment on radiological follow up. Equal numbers of BRAF-positive and BRAF-negative patients showed response rates to SRS. The remaining patients showed either brain progression or died before follow up. The majority of patients treated with WBRT alone died within 2 months of treatment, so radiological follow up was not available to assess treatment responses.

When compared with WBRT there was a statistically significant improvement in survival in patients suitable for SRS. Patients treated with SRS and/or surgery and/or systemic treatment (n = 16) had a median survival of 13.5 months (95% CI 5.93–21.1) compared to 2.2 months (95% CI 1.55–2.85) in the WBRT group (n = 21). This difference reached statistical significance (HR 3.97, p = 0.0009).

**Fig. 2:**
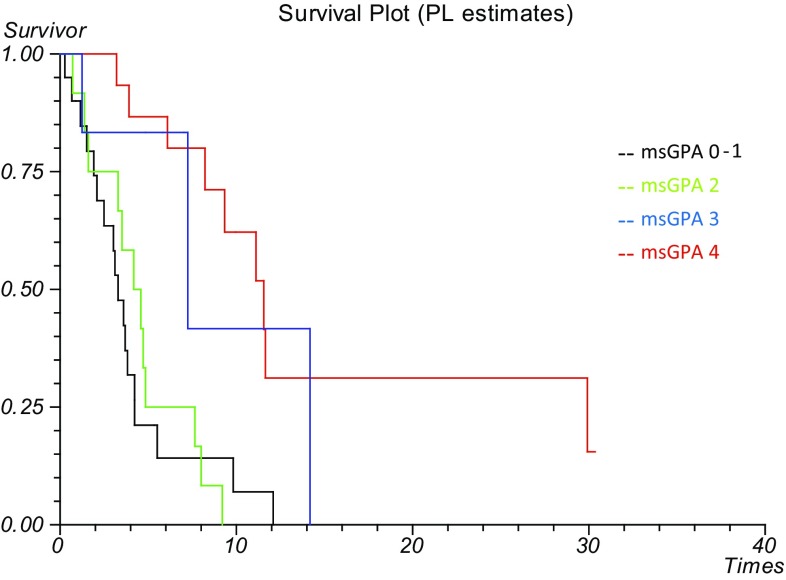
Overall survival from date of brain metastases diagnosis according to melanoma specific graded prognostic assessment score

Figure 2 shows survival outcomes based on msGPA score. Median survival was 11.6 months for those with a maximum GPA score of 4 (n = 15, 95% CI 9.29–13.84), 7.2 months for GPA 3 (n = 6, 95% CI 6.9–7.57), 4.2 months for GPA 2 (n = 12, 95% CI 2.36–5.98) and 3.3 months for GPA 0–1 (n = 20, 95% CI 2.47–4.07). This suggests that the msGPA provides a reliable indication of likely prognosis, with those scoring more highly having better survival outcomes (peto’s log rank test, p < 0.0001).

On univariate analysis, factors shown to be predictive of OS were BRAF status, KPS and msGPA score (msGPA 0–2 vs. msGPS 3–4) (Table 2). Age, sex, presence of neurological symptoms and presence of extra cranial disease were not predictors of survival on univariate analysis. Only msGPA score maintained significance on multivariate analysis (Table 3).

**Table 2:** Univariate analysis of prognostic factors

	Hazard ratio	95% CI	p-value
BRAF status			
Positive	0.345	0.163–0.732	0.0056
Negative	1		
msGPA score			
0–2	1		
3–4	0.194	0.0882–0.428	<0.0001
Neurological symptoms			
Present	1.85	0.701–4.88	0.2141
Absent	1		
Age	1.022	0.996–1.048	0.0928
Sex			
Male	1		
Female	0.513	0.261–1.012	0.0541
KPS			
70–100	1		
<70	4.003	1.56–10.26	0.0039
Extra cranial disease			
Present	1		
Absent	0.912	0.397–2.10	0.8303
Development of brain mets			
Presentation of metastatic disease	0.698	0.356–1.37	0.2941
During treatment for metastatic disease	1		

**Table 3:** Multivariate analysis of prognostic factors

	Hazard ratio	95% CI	p-value
BRAF status			
Positive	0.544	0.247–1.195	0.1294
Negative	1		
msGPA			
0–2	1		
3–4	0.238	0.105–0.539	0.0006
KPS			
70–100	1		
<70	2.178	0.832–5.704	0.1131

## Discussion

This retrospective study confirms the overall poor prognosis of patients with brain metastases from melanoma. However, it highlights certain treatment options that may be helpful such as BRAF inhibitors in BRAF mutated melanoma and SRS alone or combined with systemic treatment for suitable patients. The ability of the msGPA to accurately discriminate prognostic groupings, and thus its suitability for continued use in a modern population with access to novel treatments, is confirmed. And importantly we recommend that if WBRT alone is the only treatment option available for a particular patient this should be offered only after careful consideration and discussion with the patient as there is a very poor outlook in this group of patients.

We report a median OS from brain metastases diagnosis of 4.83 months. This is comparable other reports in the literature [4]. Patients in our cohort were almost equally distributed between mutant-positive (n = 25) and mutant-negative (n = 22) categories. This proportion is higher than commonly quoted in the literature, where BRAF mutant-positive melanomas are reported to comprise around 40% of the total melanoma population [20]. This adds weight to the growing evidence base demonstrating that BRAF-positive melanoma patients are at increased risk of development of brain metastases [21–23].

In our series the BRAF-mutant positive patients had significantly better survival times than the BRAF-mutant negative group, at 8.23 month median OS (95% CI 3.62–12.84) and 3.7 months (95% CI 2.78–4.62) respectively (p = 0.0039) from the time of diagnosis of brain metastases. This is due to the benefit of BRAF inhibitors as a treatment for brain metastases in BRAF mutation positive melanoma. The OS of our group of BRAF positive patients compares favorably to those treated in the original BREAK-MB trial, which documented survival times of up to 7.7 months [24]. Thus we provide evidence to support the efficacy of targeted therapies in producing improved survival outcomes in ‘real life’ BRAF-positive patients. BRAF status was an independent predictor of survival in univariate analysis, in concordance with previous documentation in the literature [2, 21]. It may have potential to be incorporated into prognostic modelling for brain metastases.

We assessed the use of radiotherapy for the treatment of brain metastases from metastatic melanoma; SRS combined with other systemic treatments and WBRT alone. We found evidence to support the efficacy of SRS. N = 16 (30%) patients received SRS for up to three small volume intracerebral metastases, either at first presentation of brain disease or on progression. The median survival time from treatment was 13.5 months (95% CI 5.93–21.1). This is consistent with previous literature, demonstrating increased intracranial control, OS and the ability to use multiple courses of SRS on disease progression [13–15, 25]. It has been hypothesised that BRAF-mutant positive patients may have increased responsiveness to SRS [26]. However, we found no difference in response rates according to BRAF status.

Evidence for the role of WBRT in local treatment of brain metastases from melanoma is less consistent. Many studies report no benefit to overall survival [7–9], although some neurological symptoms palliation may be offered for symptomatic patients. Others suggest that certain circumstances, such as stable extra-cranial disease or adjuvant treatment with SRS or neurosurgery, may enable WBRT to control intracranial disease for a limited period of time [11]. In our cohort, 21 patients underwent WBRT as the sole treatment for their brain metastases. Median OS was poor at just 2.2 months (95% CI 1.55–2.85). This may partially be due to an underlying selection bias for a patient population with particularly poor prognoses since WBRT was often offered to those who had exhausted systemic treatment options with multiple disseminated lesions as a palliative measure. It brings into question the appropriateness of recommending this treatment where little survival benefit must be balanced against a potential side effect profile that includes alopecia, neurocognitive decline and fatigue.

We found evidence to support the continued use of the msGPA in the modern day population with use of novel therapies. Our cohort was well distributed between the four prognostic groupings. The scoring system discriminated prognosis well, with a median survival of 11.6 months (95% CI 9.29–13.84) for those with a maximum GPA of 4, contrasted to a median survival of 3.3 months (95% CI 2.47–4.07) for those with a GPA score of 0–1. The log rank test demonstrated statistical significance in median survival outcomes between the four groupings (p < 0.0001). Our results are similar to those reported in the original Sperduto et al. study of 3.38, 4.7, 8.8 and 13.2 months with increasing msGPA score [12]. Furthermore, the msGPA demonstrated significance when tested on both univariate (p < 0.0001) and multivariate analysis (p = 0.0006) in our cohort.

We believe that our patients, recruited from 2011 to 2016, with access to novel systemic therapies, is representative of the current modern day population and practice. Thus our data demonstrates that the msGPA score is still important in modern day practice, and is able to provide patients with a reliable indication of likely prognosis.

Our results must be interpreted in the light of several limitations; firstly, the retrospective nature of the study meant that KPS scores (and therefore msGPA calculations) along with the precise number and volume of brain metastases may be less reliable. The rapidly changing landscape of melanoma treatments over recent years means that not all the patients in our sample had access to all the treatment options in the same sequence. For example, during the period studied PD1 inhibitors were available in the second line setting only initially and then subsequently as first line. The sample size of 53 patients limits the power of the study to produce statistically significant results applicable outside our cohort. We suggest that further studies of similar design, on a larger cohort, be executed to test the validity of our observations.

## Conclusion

Our study has provided evidence to validate the use of the msGPA in the modern patient population, with its access to novel treatments. The system provides clinicians with an indication of prognosis and may aid patient centered treatment discussions. We have demonstrated that BRAF positive patients receiving targeted treatment have significantly better survival than their BRAF negative counterparts. We were able to evidence the efficacy of SRS in the local treatment of brain metastases irrespective of BRAF status. By contrast, the suitability of WBRT as a stand-alone management option has been called in to question. Patients unsuitable for SRS who therefore received WBRT had a very poor outcome.
